# Association Between Serum 25-OH-Vitamin D and Diabetic Foot Ulcer in Patients With Type 2 Diabetes

**DOI:** 10.3389/fnut.2020.00109

**Published:** 2020-09-02

**Authors:** Jiezhi Dai, Min Yu, Hua Chen, Yimin Chai

**Affiliations:** ^1^Department of Orthopedic Surgery, Shanghai Jiao Tong University Affiliated Sixth People's Hospital, Shanghai, China; ^2^Department of Vascular Surgery, Longhua Hospital Shanghai University of Traditional Chinese Medicine, Shanghai, China

**Keywords:** diabetic foot ulcer, 25-OH-vitamin D, patients with diabetes, vitamin D deficiency, association

## Abstract

**Background:** Vitamin D deficiency has been associated with an increased risk in several diabetic complications. We aimed to evaluate the association between vitamin D and diabetic foot ulcer (DFU) in patients with type 2 diabetes.

**Methods:** Fifty one patients were included in the study and divided into two groups for study of vitamin D, cholesterol, and triglycerides in blood serum on DFU. The association between vitamin D and DFU was measured by binary logistic regression analysis. The cut point of vitamin D for DFU was assessed by the receiver operating characteristic curve.

**Results:** Levels of 25-OH-vitamin D were lower in patients with DFU than in DM group (*P* < 0.0001). The AUC of 25-OH-vitamin D was 0.8254 and had an optimal cut point value (13.68 ng/ml) for the identification of DFU, with a sensitivity of 90% and a specificity of 66.67% in all patients. Multivariate logistic regression analysis indicated that the significant risk factors included 25-OH-vitamin D level (*P* = 0.001, OR = 0.618) and HDL-C level (*P* = 0.038, OR = 0.012).

**Conclusion:** Low serum 25-OH-vitamin D level was associated with DFU. This indication was more specific than cholesterol and triglycerides levels.

The diabetic foot ulcer (DFU) is a common complication of diabetes mellitus (1). Poor wound healing leads to high hospitalization rate and increases the probability of lower extremity amputation (2). It is associated with a substantial decrease in quality of life and increased risk of morbidity and mortality. Chammas et al. (3) reported that DFU patients had a >2-fold increase in mortality compared with non-ulcerated diabetic patients.

Vitamin D is a pleiotropic steroid hormone that has multiple biologic effects. It is integral to the regulation of calcium homeostasis and bone turnover as and is known to be an important immune modulator (4). Vitamin D deficiency has been found as a potential risk factor for increased cardiovascular risk, abnormal HDL cholesterol (HDL-C) and LDL cholesterol (LDL-C) levels, hypertension, hyperglycemia, and diabetes (5, 6).

In this study, we study the association between vitamin D, HDL cholesterol, LDL cholesterol, total cholesterol and triglycerides in blood serum on diabetic foot ulcer and try to investigate the prevalence of vitamin D insufficiency in patients with DFU.

## Patients and Methods

This prospective study was conducted on 51 patients including 21 patients with DFU and 30 newly admitted type 2 diabetes patients without DFU (control group), between Jan 2019 and Oct 2019 in Shanghai Jiao Tong University Affiliated Sixth People's Hospital. The study protocol was approved by the Ethic Review Board of Shanghai Six People's Hospital affiliated to Shanghai Jiao Tong University (YS-2019-010). Written informed consent was obtained from all of the enrolled participants. The study was conducted in accordance with the principles of the Declaration of Helsinki. Patients who were diagnosed with type 2 diabetes based on the diagnostic criteria recommended by the ADA in 2010 were included (7). Patient with type 1 diabetes, thyroid disorders, rheumatologic, serious hepatic, cardiac, renal failure, and malignancy were excluded (8). We also excluded pregnant women and patients with vitamin D supplement consumption over the past 6 months (9). DFU is defined as “ulceration of the foot (distally from the ankle and including the ankle) associated with neuropathy and different grades of ischemia and infection” according to the World Health Organization (10). We excluded chronic wound caused by pressure ulcer, vasculitis, pyoderma gangrenosum, and diseases that cause ischemia (11).

Patient's age, sex, body mass index (BMI), duration of diabetes and the degree of the ulcer were recorded. Blood samples were taken in the morning after overnight fasting. The following parameters were measured in both groups: fasting blood glucose (FBG), HbA1c, high-sensitive C-reactive protein (hs-CRP), erythrocyte sedimentation rate (ESR), 25-OH-vitamin D, HDL-C, LDL-C, Total Cholesterol (TC), and Triglyceride (TG). FBG was measured by glucose oxidase method. HbA1c was assayed using the chromatography. 25-Hydroxyvitamin D was determined with a commercially available radioimmunoassay. hs-CRP was measured by high sensitive nephelometric assay. ESR was tested by TEST-1 Analyzer. HDL-C, LDL-C, TC, and TG were measured with enzymatic method. The University of Texas Wound Classification System was used to grade diabetic foot ulcer (Grade I: superficial wound, not involving, tendon, capsule or bone, Grade II: wound penetrating to tendon or capsule and grade III: wound penetrating bone or joint) (12).

SPSS 18.0 software was used for statistical analyses. Data are presented as means and standard deviations. Differences between groups were tested with ANOVA tests. Receiver operating characteristic (ROC) curve analysis was performed to reveal the sensitivity and specificity of 25-OH-vitamin D, HDL-C, LDL-C, TC, and TG for predicting DFU. The cut-off values were from the ROC curves. The area under the curve (AUC) was used to assess the diagnostic accuracy. The association between 25-OH-vitamin D and clinical characteristics was performed by Spearman's correlation analysis. A logistic forward regression analysis was used to identify the association between the variables. The level of statistical significance was 0.05.

## Results

Age, gender, duration of diabetes, BMI, levels of FBG, and HbA1c did not differ between the patients groups (Table 1). Levels of 25-OH-vitamin D were lower in patients with DFU than in DM group (*P* < 0.0001). Inflammatory markers including hs-CRP and ESR were significantly higher in the group with DFU when compared with the DM group (*P* < 0.0001 and < 0.0001, respectively). HDL cholesterol levels in DFU group were significantly lower when compared with in the DM group (*P* = 0.015). In 21 patients with DFU, all the patients had 25-OH-vitamin D deficiency with values below 50 nmol/L (20 ng/ml), and nine patients had severe 25-OH-vitamin D deficiency with values below 25 nmol/L (10 ng/ml). In 21 patients with DFU, all the patients had 25-OH-vitamin D deficiency with values below 50 nmol/L (20 ng/ml), and nine patients had severe 25-OH-vitamin D deficiency with values below 25 nmol/L (10 ng/ml). In 30 patients with DM, 23 patients (76.67%) had 25-OH-vitamin D deficiency with values below 50 nmol/L (20 ng/ml), and none of patients had severe 25-OH-vitamin D deficiency with values below 25 nmol/L (10 ng/ml). The degrees of ulcer used the University of Texas Wound Classification System. Six patients were grade I, 12 patients were grade II and three patients were grade III.

**Table 1:** Clinical characteristics of the study.

	**Study group**	**Control group**	***P***
N (male/female)	21 (13/8)	30 (16/14)	
Age (ys)	62.00 ± 5.93	59.23 ± 5.70	0.100
Duration (ys)	11.00 ± 3.70	10.03 ± 4.38	0.413
BMI (kg/m^2^)	23.74 ± 3.05	25.55 ± 3.70	0.088
FBG (mmol/L)	7.28 ± 1.98	7.24 ± 1.38	0.923
HbA1c (%)	8.60 ± 2.41	8.39 ± 1.36	0.691
hs-CRP (mg/L)	35.46 ± 43.05	0.89 ± 1.09	<0.0001
ESR (mm/h)	67.81 ± 29.56	22.4 ± 10.45	<0.0001
Vitamin D level (ng/ml)	11.21 ± 5.20	17.73 ± 3.20	<0.0001
TC (mmol/L)	4.07 ± 1.40	4.51 ± 0.90	0.178
TG (mmol/L)	1.35 ± 0.69	1.79 ± 0.99	0.084
HDL-C (mmol/L)	0.90 ± 0.30	1.11 ± 0.27	0.015
LDL-C (mmol/L)	2.58 ± 0.97	2.82 ± 0.82	0.352

Spearman's correlation analysis showed that age was correlated with the levels of 25-OH-vitamin D (*P* = 0.026, *r* = 0.485) (Table 2). There was no correlation between the vitamin D levels and duration, BMI, FBG, HbA1c, hs-CRP, ESR, TG, TC, HDL-C, or LDL-C, respectively.

**Table 2:** Spearman's correlation coefficients of 25-OH-vitamin D with Age, Duration, BMI, FBG, HbA1c, TG, TC, HDL-C, and LDL-C.

	**25-OH-vitamin D**	
	**γ**	***P***
Age (ys)	0.485	0.026
Duration (ys)	−0.123	0.595
BMI (kg/m^2^)	0.352	0.118
FBG (mmol/L)	−0.161	0.487
HbA1c (%)	−0.047	0.840
hs-CRP (mg/L)	−0.336	0.136
ESR (mm/h)	0.088	0.705
TC (mmol/L)	0.255	0.265
TG (mmol/L)	0.047	0.838
HDL-C (mmol/L)	0.255	0.264
LDL-C (mmol/L)	0.375	0.094

**Figure 1:**
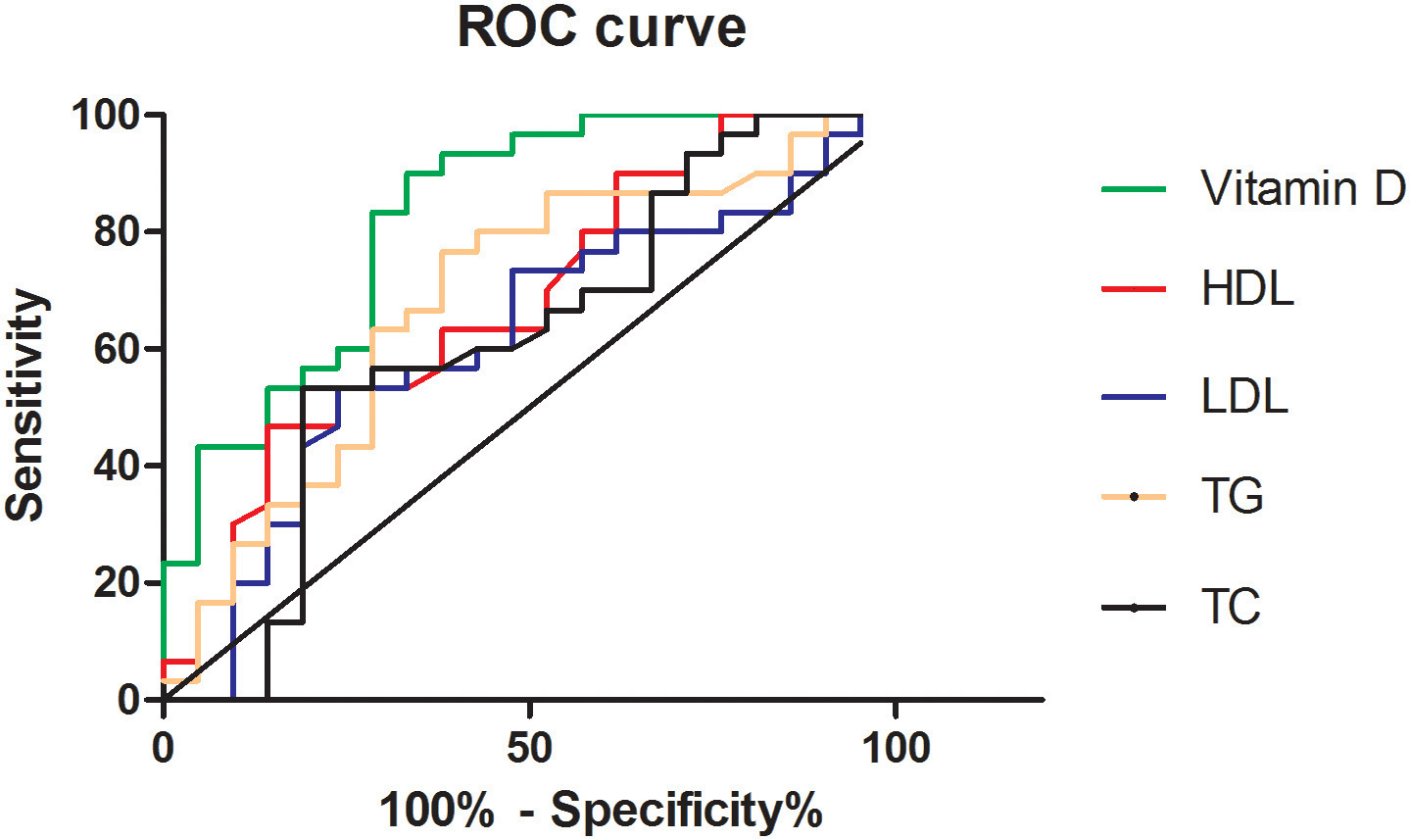
ROC analysis of vitamin D, HDL-C, LDL-C, TC, and TG to indicate diabetic foot ulcer.

ROC analysis was performed to reveal the diagnostic accuracy of using 25-OH-vitamin D, HDL-C, LDL-C, TG, and TC for DFU. Sensitivity, specificity, the best cutoff value, and the AUC were presented in Table 3 and Figure 1. A cut-off value was taken when sensitivity and specificity got balanced in our study. The AUC of 25-OH-vitamin D was 0.8254 (95% CI, 0.7068 to 0.9440) and had an optimal cut point value (13.68 ng/ml) for the identification of DFU, with a sensitivity of 90% and a specificity of 66.67% in all patients.

**Table 3:** The specificity, sensitivity, best cut-off value, and the area under the curve of Vitamin D, HDL-C, and LDL-C in patients with DFU.

**Parameter**	**AUC**	***P***	**95% CI**		**Cut-off value**	**Sensitivity%**	**Specificity%**
			**Lower**	**Upper**			
Vitamin D ng/ml	0.8254	<0.0001	0.7068	0.9440	13.68	90	66.67
HDL-C mmol/L	0.6794	0.0306	0.5289	0.8298	0.81	90	38.10
LDL-C mmol/L	0.6151	0.1653	0.4549	0.7752	2.945	53.33	76.19
TC mmol/L	0.6175	0.1568	0.4513	0.7836	4.915	53.33	80.95
TG mmol/L	0.6833	0.0271	0.5300	0.8367	1.215	76.67	61.90

Associations of vitamin D, HDL-C, LDL-C, TC, and TG with the dependent variable DFU were explored in the logistic regression analysis (Table 4). The results showed that the significant risk factors for DFU included 25-OH-vitamin D level (*P* = 0.001, OR = 0.618) and HDL-C level (*P* = 0.038, OR = 0.012).

**Table 4:** Logistic regression predicting likelihood of DFU on vitamin D, HDL-C, LDL-C, TC, and TG.

	**B**	**S.E**.	**Wald**	**df**	***P***	**OR**	**95% CI for OR**	
							**Lower**	**Upper**
Vitamin D	−0.482	0.147	10.740	1	0.001	0.618	0.463	0.824
HDL-C	−4.409	2.124	4.310	1	0.038	0.012	0.000	0.782
LDL-C	2.179	1.232	3.130	1	0.077	8.838	0.791	98.784
TC	−1.297	0.909	2.036	1	0.154	0.273	0.046	1.624
TG	−1.316	0.724	3.307	1	0.069	0.268	0.065	1.108

## Discussion

DFU is an important complication of diabetes. It is often characterized by severe infections and associated with significant morbidity and mortality, which having immense social, psychological, and financial consequences (13). Therefore, the early identification of diabetic subjects at high risk for foot ulceration could permit the prevention of their severe complications (14).

A meta-analysis by Pei et al. (15) assessed the effect of lipids and lipoproteins on the development of diabetic foot in patients with DM. The authors concluded that reduced HDL cholesterol levels not LDL-C, TC, or TG levels were associated with DFU. Ansell et al. (16) reported the anti-inflammatory effects of HDL cholesterol with a decrease in inflammatory cytokines and vascular leukocyte adhesion molecules, participation in innate immunity and prevention of LDL oxidation. Similar findings have been demonstrated in our study. We also reported a lower level of HDL cholesterol levels in DFU group when compared with DM group.

In recent years, observational association between vitamin D deficiency and diabetes mellitus has been well-discussed (17). Yoho et al. (18) reported vitamin D levels were significantly lower in the diabetic populations compared to the non-diabetic control group. Serum vitamin D levels have been shown to improve glycemic control and vitamin D supplementation was found to decrease HbA1c level in diabetic patients (19, 20). Some research have reported its effect on T cell mediated immunity, pancreatic insulin secretion and action as well as cell growth and healing (21). A randomized, double-blind, placebo-controlled trial, by Razzaghi et al., showed that vitamin D, compared with placebo, entailed a more significant improvement in wound parameters (22). We also conducted a meta-analysis to evaluate the association between vitamin D deficiency and DFU and reported a significantly reduction of vitamin D levels in DFU group when compared with DM group (23). In this study, we study the association between vitamin D, HDL-C, LDL-C, TG, and TC in blood serum on diabetic foot ulcer. We would like to add that there are, apart from lipids, other markers of vascular risk which may have a bearing on the incidence of DFU.

This study involving 51 patients investigated the relationship between 25-OH-vitamin D and DFU. 25-OH-vitamin D levels in DFU group were significantly lower than DM group. This result revealed that 25-OH-vitamin D had the highest AUC and the greatest statistical significance in the DFU group. The area under the ROC curve for 25-OH-vitamin D was the greatest (0.8254), followed by TG (0.6833) and HDL-C (0.6794), and in the end, by TC (0.6175) and LDL-C (0.6151). We concluded that vitamin D can be more helpful in the diagnosis of DFU when compared with HDL cholesterol.

Several limitations were found in our study. First, only fifty patients were included and the number was relatively small. Second, the molecular mechanisms underlying the role of vitamin D in wound healing in diabetic patients remained poorly known. Further well-designed researches are needed in the future.

In the current study, we demonstrated the associations of vitamin D with DFU. This indication was more specific than cholesterol and triglycerides levels. The present study might be helpful in early prevention and diagnosis of DFU in diabetic patients.

## Data Availability Statement

The datasets generated for this study are available on request to the corresponding authors.

## Ethics Statement

The studies involving human participants were reviewed and approved by the Ethic Review Board of Shanghai Six People's Hospital affiliated to Shanghai Jiao Tong University. The patients/participants provided their written informed consent to participate in this study. Written informed consent was obtained from the individual(s) for the publication of any potentially identifiable images or data included in this article.

## Author Contributions

HC: conceptualization. JD: investigation. MY: methodology. JD: writing—original draft. HC and YC: writing—review and editing. All authors contributed to the article and approved the submitted version.

## Conflict of Interest

The authors declare that the research was conducted in the absence of any commercial or financial relationships that could be construed as a potential conflict of interest.
